# Successfully treated infected aneurysm caused by *Listeria monocytogenes*

**DOI:** 10.1016/j.idcr.2021.e01206

**Published:** 2021-06-26

**Authors:** Hirokazu Toyoshima, Koji Hirano, Motoaki Tanigawa, Naoto Masuda, Chiaki Ishiguro, Hiroyuki Tanaka, Yuki Nakanishi, Shigetoshi Sakabe

**Affiliations:** a Department of Infectious Diseases, Japanese Red Cross Ise Hospital, Ise 516-8512, Japan; b Department of Thoracic and Cardiovascular Surgery, Japanese Red Cross Ise Hospital, Ise 516-8512, Japan; c Department of Respiratory Medicine, Japanese Red Cross Ise Hospital, Ise 516-8512, Japan; d Department of Medical Technology, Japanese Red Cross Ise Hospital, Ise 516-8512, Japan

**Keywords:** *Listeria monocytogenes*, Infected aneurysm, Mycotic aneurysm, Rapid diagnostic testing, In-situ graft placement

## Abstract

Infected aneurysms caused by *Listeria monocytogenes* are extremely rare. Therefore, there is no standard procedure for their diagnosis and treatment. A 76-year-old Japanese man with diabetes and hypertension was diagnosed with a left common iliac aneurysm caused by *L. monocytogenes*, using multidetector computed tomographic angiography and rapid diagnostic testing of the positive blood culture. He was successfully treated with a combination of ampicillin administration, timely surgical debridement, and in-situ Y-graft placement with revascularization and omental implantation. Vancomycin and third-generation cephalosporins, to which *L. monocytogenes* is resistant, are used as an empirical regimen for infected aneurysms. Therefore, the use of a rapid diagnostic testing is important as it identifies *L. monocytogenes* within 24 h from obtaining the blood cultures, and guides the administration of the appropriate antibiotics. In-situ Y-graft placement restores nearly normal blood flow, following the confirmation of negative conversion of blood culture in response to the intensive intravenous ampicillin therapy. Appropriate and timely microbiological examinations, in addition to radiographic examinations, can be the key for selecting the optimal therapeutic procedures for each patient and achieving the best possible outcomes.

## Highlights

• *L. monocytogenes* causes seriously infected aneurysms in immunocompromised hosts.
• Cephalosporins are used as an empirical therapy for infected aneurysms.
• *L. monocytogenes* has intrinsic resistance to cephalosporins.
• Rapid diagnostic testing is helpful in patients with life-threatening listeriosis.
• A short course of preoperative therapy with appropriate antibiotic is beneficial.

## Introduction

*Listeria monocytogenes* is a food-borne pathogen, which causes rare but serious infections, particularly in the elderly, pregnant women, and newborn infants, owing to their compromised immune system [1]. The incidence rate of *L. monocytogenes*-infected aneurysms has been increasing since the 2000s, particularly among older men with immunosuppressive comorbidities [1,2]. The serious infections caused by *L. monocytogenes* are divided into three major types, namely maternal infection, bacteremia, and neurolisteriosis [2], and the classification is based on the location of the infection, including peritoneal, bones and joints, pleural, cardiovascular, urinary, biliary tract, lungs, and lymph glands [2]. Infected aneurysms are commonly caused by *Staphylococcus* and *Salmonella*, among other species [3]. Among these, *L. monocytogenes*-infected aneurysms are life threatening; however, there are no clinical trials and established treatment protocols, because of their rarity [4]. Patients with infected aneurysms and undergoing only medical treatment have a high rate of mortality, ranging between 75 % and 100 %, resulting from aneurysmal rupture [5].

Here, we describe a rapidly diagnosed and successfully treated case of *L. monocytogenes*-infected aneurysm in a 76-year-old man. This report emphasizes that rapid diagnosis and intensive treatment guided by imaging, molecular analysis, and in-situ graft placement can save patients with *L. monocytogenes*-infected aneurysms.

## Case

A 76-year-old Japanese man with diabetes and hypertension presented with left lower quadrant abdominal and medial femoral pain for the previous 10 days. He was diagnosed with a left common iliac aneurysm by the consultant who referred him to our hospital. He denied any gastrointestinal symptoms before admission. He had no known allergies and was prescribed angiotensin II receptor blocker for hypertension and insulin for diabetes; however, he had discontinued these medications two months before hospitalization based on his own judgment.

He was alert (Glasgow Coma Scale 15), and his vital signs were as follows: body temperature, 38.2 °C; blood pressure, 117/54 mmHg; heart rate, 67 beats/min; respiratory rate, 14 breaths/min; and oxygen saturation, 98 % on ambient air. Physical examination indicated mild tenderness on the left lower abdomen. The findings from other examinations, including the palpation of bilateral dorsalis pedis arteries, were normal.

The laboratory findings were as follows: albumin, 3.0 g/dL; alanine transferase, 11 U/L; aspartate aminotransferase, 15 U/L; lactate dehydrogenase, 139 U/L; creatine kinase, 30 U/L; lactate, 7.7 mg/dL; blood urea nitrogen, 17 mg/dL; creatinine, 0.94 mg/dL; C-reactive protein, 5.15 mg/dL; blood glucose, 155 mg/dL; hemoglobin A1c (HbA1c), 6.9 %; white blood cell count, 6400/μL with 68.7 % neutrophils; hemoglobin, 8.8 g/dL; and platelet count, 22.2 × 10^4^/μL.

**Fig. 1:**
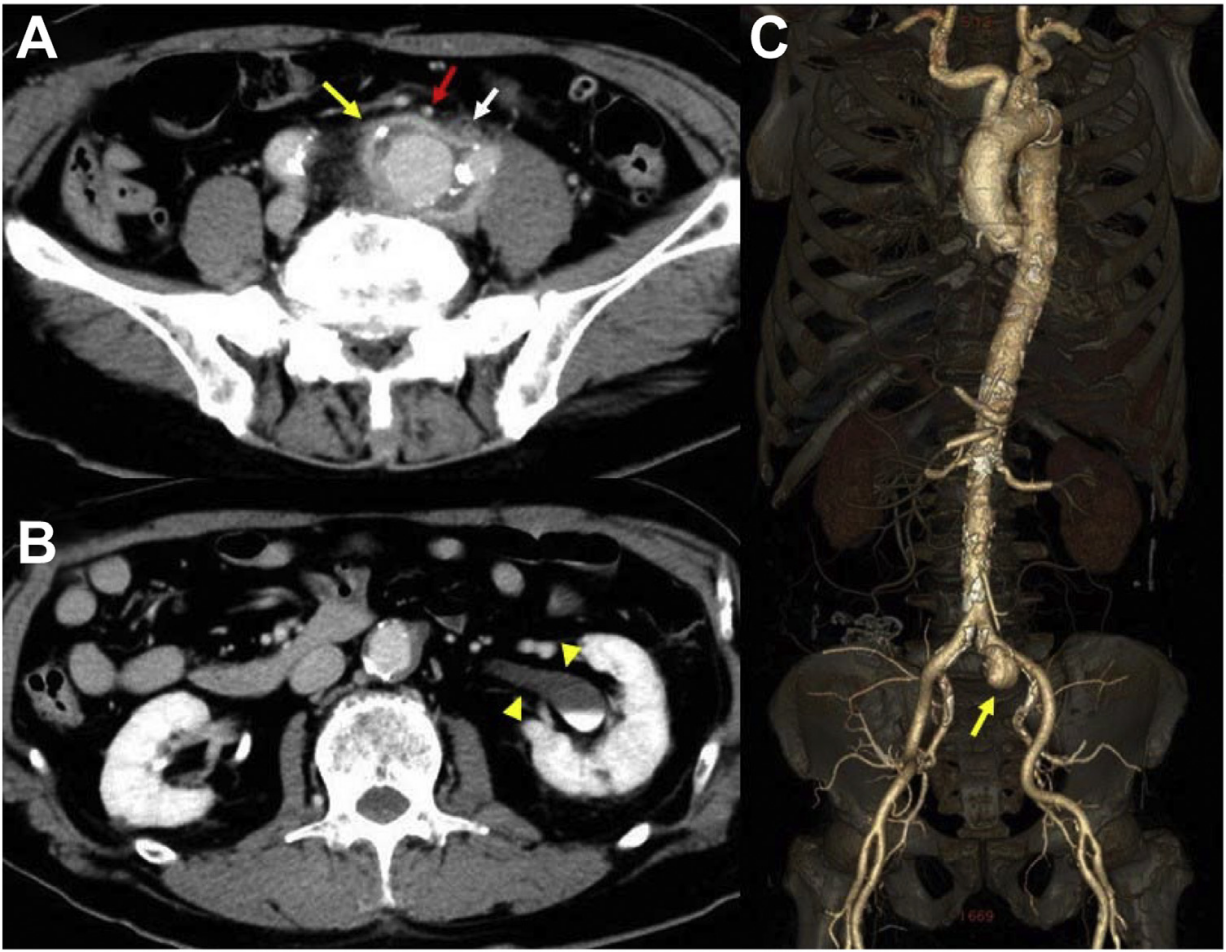
Enhanced abdominal computed tomography (CT) (A, B) and multidetector computed tomographic angiography (C) findings showed a 27 × 32 mm sized saccular pseudoaneurysm (yellow arrows) with the surrounding inflammation, involving inferior mesenteric artery (red arrow) and left ureter (white arrow), in the left common iliac artery (A, C). The compression of the left ureter led to left hydronephrosis (yellow arrowheads) (B). (For interpretation of the references to colour in this figure legend, the reader is referred to the web version of this article).

Abdominal computed tomography (CT) showed a 27 × 32 mm sized saccular pseudoaneurysm surrounded by inflammation in the left common iliac artery (Fig. 1; A, C). The inflammation affected the inferior mesenteric artery and the left ureter (Fig. 1A), which caused left hydronephrosis (Fig. 1B).

**Fig. 2:**
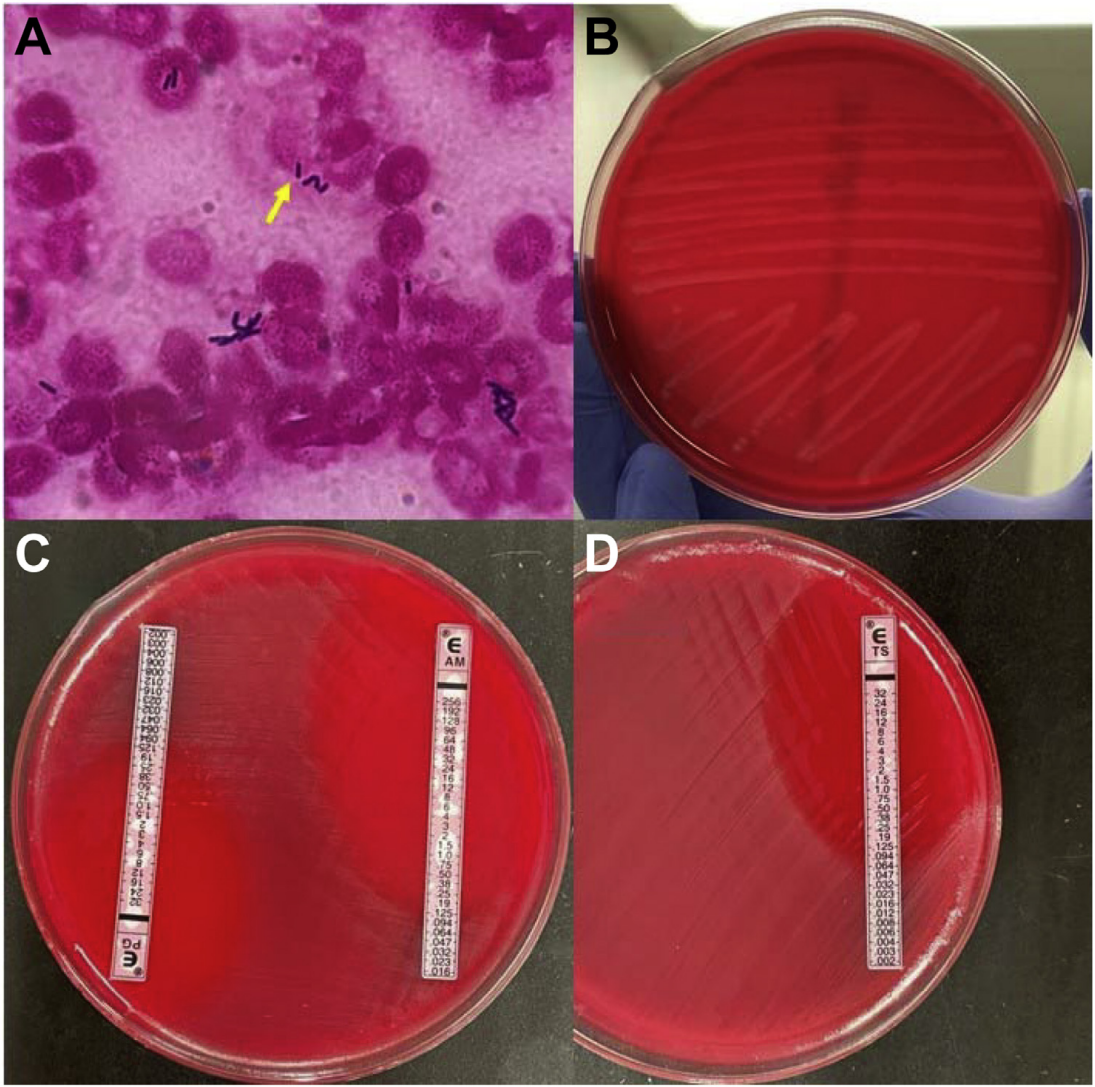
Microbiological findings. Gram staining (×1000) revealed Gram-positive rods (yellow arrow) (A). The colonies showed a narrow zone of beta-hemolysis (B). E-test® (bioMérieux) showed penicillin, ampicillin, and sulfamethoxazole/trimethoprim to have minimum inhibitory concentrations (MICs) of 0.19, 0.125, and 0.064 μg/mL, respectively (C, D). (For interpretation of the references to colour in this figure legend, the reader is referred to the web version of this article).

Two sets of aerobic and anaerobic blood cultures (BacT/Alert FAN plus, bioMérieux, Marcy I’Etoile, France) obtained at admission turned positive within 21 h. Gram staining showed Gram-positive rods (Fig. 2A), which were quickly and accurately identified as *L. monocytogenes*, using the matrix-assisted laser desorption ionization (MALDI) biotyper Sepsityper kit® (Bruker Daltonik GmbH, Bremen, Germany) with a high score value (2.03) and the Verigene® system (Luminex, Austin, Texas, USA), within 24 h of obtaining the blood cultures. Isolates grew on 5% sheep blood agar (Nihon Becton-Dickinson, Tokyo, Japan), following 24 h incubation at 5% CO_2_ and 37 °C and exhibited a narrow zone of beta-hemolysis (Fig. 2B). The E-test® (bioMérieux, Marcy I’Etoile, France) suggested minimum inhibitory concentrations (MICs) of 0.19, 0.125, and 0.064 μg/mL for penicillin, ampicillin, and sulfamethoxazole/trimethoprim, respectively, which indicated susceptibility, according to the Clinical and Laboratory Standards Institute (CLSI) document (M100-Ed30) (Fig. 2; C, D) [6]. There was no evidence of infective endocarditis, as evaluated by transthoracic and transesophageal echocardiography or bacterial meningitis, as observed from the cerebrospinal fluid examination on day 2. In addition, the stool culture obtained during admission was negative for *L. monocytogenes*.

**Fig. 3:**
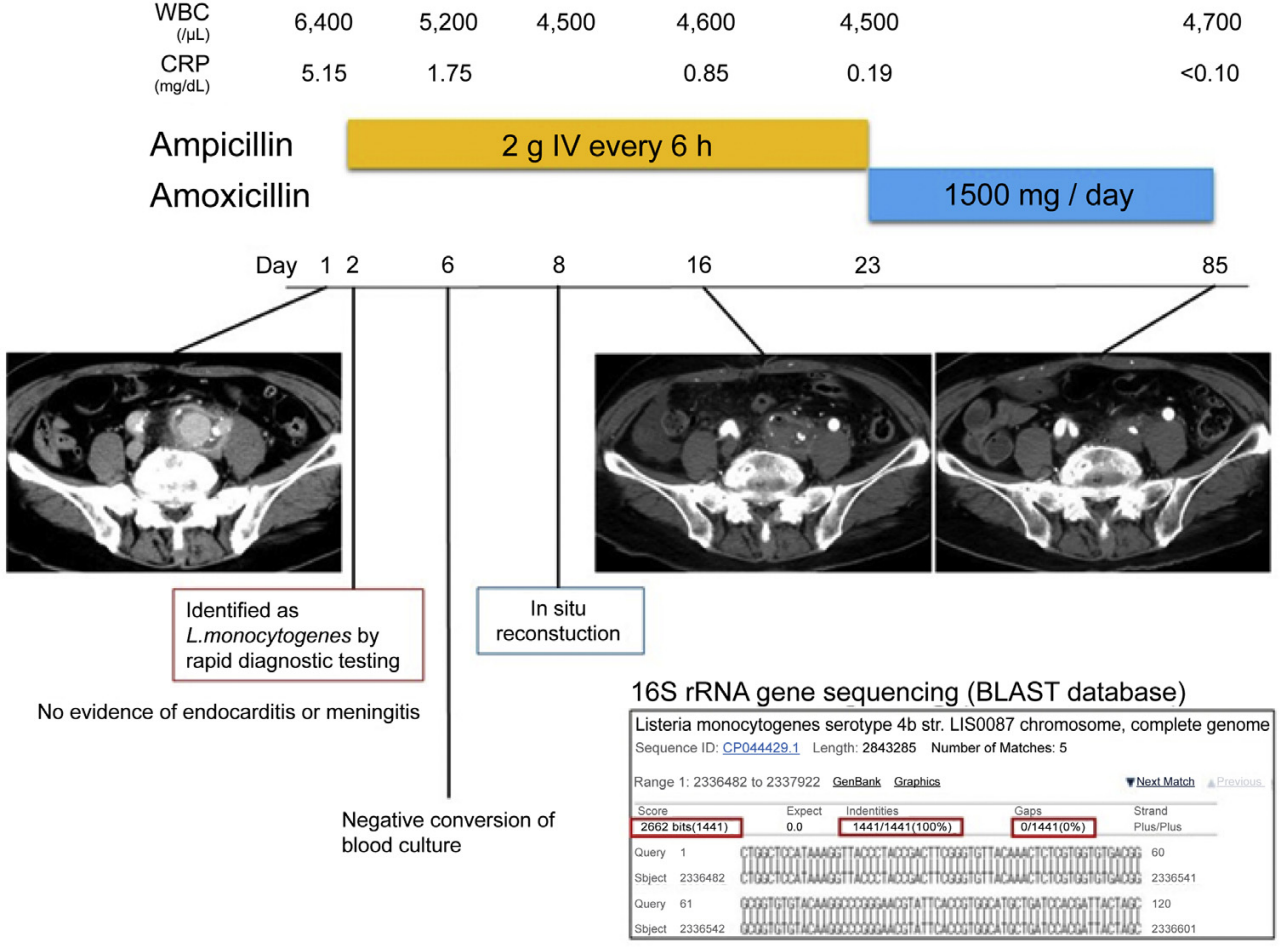
Clinical course of the patient. The patient was diagnosed with *Listeria monocytogenes*-infected aneurysm using the rapid diagnostic testing and the multidetector computed tomographic angiography finding, in addition to blood culture results within 24 h. Antibiotic therapy, using ampicillin was administrated on day 2 and blood pressure was well-controlled. There was no evidence of infective endocarditis or bacterial meningitis based on transthoracic and transesophageal echocardiography, and cerebrospinal fluid examination, respectively. Repeated blood cultures on day 6 tested negative for *L. monocytogenes*. In-situ Y-graft (Japan Lifeline Co.) placement with revascularization and omental implantation was performed on day 8. The isolate was consistent with *L. monocytogenes* serotype 4b, as determined using 16S rRNA gene sequencing and the GenBank Basic Local Alignment Search Tool (BLAST) analysis (www.ncbi.nlm.nih.gov/genbank/) (Identities 1441/1441, Gaps 0/1441, Score 2662bits). After three weeks of intravenous antibiotic therapy, the intravenous ampicillin was replaced with oral amoxicillin. Oral amoxicillin was discontinued on day 85 since all anastomoses remained patent without left hydronephrosis in the follow-up CT. The patient has remained disease-free without recurrence.

The patient was diagnosed with an infected aneurysm caused by *L. monocytogenes* and treated with 2 g of intravenous ampicillin every 6 h. He became apyrexial without any massive rupture of the infected aneurysm, because of the administration of the appropriate antibiotic; his blood pressure was well controlled with an intravenous antihypertensive calcium channel blocker. Blood cultures from samples obtained on day 6 were negative for *L. monocytogenes*. Therefore, surgical debridement, followed by in-situ Y-graft (Japan Lifeline Co., Ltd., Tokyo, Japan) placement with revascularization including the inferior mesenteric artery and omental implantation, was performed on day 8. The resected specimen culture was negative for *L. monocytogenes*, which could be attributed to the intensive antibiotic therapy before the surgery. Postoperative CT on day 16 indicated that all the anastomoses were patent without any complications (Fig. 3). After three weeks, the antibiotic therapy was changed from intravenous ampicillin to oral amoxicillin and the patient was discharged on day 23. Oral amoxicillin was discontinued on day 85, when all anastomoses remained patent without left hydronephrosis in the follow-up CT, and the patient has remained disease-free without recurrence (Fig. 3).

## Discussion

This report highlights two clinical strategies that could be critical for the diagnosis and treatment of *L. monocytogenes*-infected aneurysms: rapid diagnostic testing and the strategy for clinical management of an infected aneurysm.

*L. monocytogenes* is a facultative, beta-hemolytic Gram-positive rod that can grow on blood agar medium under 5% CO_2_ at 37 ℃. Although it can be identified using conventional methods [7], it takes several days to obtain results in cases of serious listeriosis. As an empirical therapy, patients with infected aneurysms are treated with anti-staphylococcal agents, such as vancomycin and third-generation cephalosporins or ciprofloxacin, when suspecting *Staphylococcus* species, *Streptococcus* species, or Enterobacteriaceae (e.g., *Salmonella* species) [5]. *L. monocytogenes* has intrinsic resistance to cephalosporins; clinical failure with vancomycin or ciprofloxacin therapy has been reported [8,9]. This indicates that rapid diagnostic testing, such as using the MALDI biotyper Sepsityper kit® (Bruker Daltonik GmbH) or the Verigene® system (Luminex, Austin, Texas, USA), is helpful in patients with life-threatening listeriosis. The prospective *L. monocytogenes* isolates were identified with 100 % (1112/1112) accuracy using the Bruker Daltonics (Bremen, Germany) MALDI-time of flight mass spectrometry (MALDI-TOF MS) system [10]. Earlier, two cases with blood stream infections were diagnosed with *L. monocytogenes* bacteremia using MALDI-TOF MS-based direct identification using positive blood cultures [11]. The Verigene® system (Luminex) exhibited high sensitivity (18/18, 100 %) and specificity (1157/1157, 100 %) [12,13]. The MALDI biotyper Sepsityper kit® (Bruker Daltonik GmbH) identifies microorganisms within 30 min, and the Verigene® system (Luminex) identifies microorganisms within 150 min [12,13]. In our case, we suspected *Listeria* species, based on the Gram staining within 24 h of obtaining blood cultures; and diagnosed *L. monocytogenes*-infected aneurysm, based on the rapid diagnostic testing and multidetector computed tomographic angiography finding in addition to the clinical presentation. The effectiveness of ampicillin was confirmed using the E-test® (bioMérieux). This was followed by the surgical debridement and in-situ Y-graft (Japan Lifeline Co.) placement with revascularization and omental implantation on day 8; the blood cultures tested negative before the procedure. The isolate was consistent with *L. monocytogenes* serotype 4b based on 16S rRNA gene sequencing and the GenBank Basic Local Alignment Search Tool (BLAST) (www.ncbi.nlm.nih.gov/genbank/) (Identities 1441/1441, Gaps 0/1441, Score 2662bits) analysis (Fig. 3) [14]. *L. monocytogenes* serotype 4b includes hypervirulent clones, such as clonal complex (CC) 1, CC2, CC4, and CC6, which were isolated from 42 % of *L. monocytogenes*-infected aneurysms [4]. A gastric pH of less than 3 has a bactericidal effect on *L. monocytogenes*; however, the bacterial survival increases at pH 3.5 and even more at pH 4 [15]. Proton pump inhibitors elevate gastric pH, contributing to the increased risk for severe listeriosis [16]. Cell-mediated immunity plays an important role in alleviating *L. monocytogenes* infections, because it causes intracellular infections [17]. Our patient was prescribed a proton pump inhibitor for treating *Helicobacter pylori*, in addition to amoxicillin and clarithromycin, four weeks before admission. The presence of the hypervirulent clone, diabetes, and the prescribed proton pump inhibitor may have contributed to the onset and progression of the infected aneurysm, despite no history of any food intake that could cause a *Listeria* infection after treatment of *H. pylori* and the absence of gastrointestinal symptoms.

Aminopenicillins are a group of first-line antibiotics for treating *L. monocytogenes* infections; however, the optimal duration of preoperative antibiotic therapy varies, depending on several factors such as immunosuppressive comorbidities, urgency for rupture, and methods of the impending surgery (e.g., in-situ versus extra-anatomical reconstruction). In Taiwan, two to six weeks of preoperative antibiotic therapy is considered optimal for treating infected aneurysms, unless urgent surgery is indicated [5]. Our patient underwent intravenous administration of ampicillin as preoperative therapy, followed by in-situ reconstruction on day 8, after confirming the negative conversion of the previously obtained blood cultures, resulting in a favorable clinical course with no recurrence. In addition, the resected specimen tested negative for *L. monocytogenes*. We believe that a short course of preoperative treatment with appropriate antibiotics, under a condition of negative conversion of blood cultures, is acceptable for infected aneurysms caused by *L. monocytogenes*, which may cause prosthetic graft infections. In addition, the optimal duration of postoperative antibiotic therapy varies depending on pre-existing factors; the duration can range from nil to lifelong [5]. A continuous layer of endothelial cells lines the vascular lumen following implantation for 12 weeks [18]. *L. monocytogenes* affects prosthetic grafts and causes refractory prosthetic infections [4]. Therefore, we administered intravenous ampicillin for three weeks followed by oral amoxicillin for another nine weeks to prevent prosthetic *L. monocytogenes* infections. Our patient was disease-free with no exacerbation of graft infection after the 12-week antibiotic therapeutic course.

Clinicians need to make a choice between initial mono- and bi-antibiotic therapies for *L. monocytogenes-*infected aneurysms. A retrospective study did not show improved outcomes of aminoglycoside synergy with respect to early mortality (defined as death occurring 3–14 days after admission) [19]. The predictors included renal failure, previous corticosteroid therapy, and age (more than 65 years old) [19]. We suspected that it was difficult to adjust the blood concentration of aminoglycoside due to the left hydronephrosis caused by the infected aneurysm and because our patient (76 years old) had the potential risk of renal dysfunction due to aminoglycoside. Therefore, we adopted a mono-antibiotic regimen with only ampicillin, and the repeated blood culture obtained on day 6 tested negative. This indicates that an appropriate mono-antibiotic therapy is a promising option for treating aneurysms, while alleviating the adverse effects related to antibiotic use in the population with these predictors listed above.

Surgical intervention is required in most cases of *L. monocytogenes*-infected aneurysms due to the high mortality rate ranging between 75 % and 100 %, resulting from aneurysmal rupture in patients undergoing only medical treatment for infected aneurysms [5]. Over the past 50 years, extra-anatomical reconstruction has been the standard surgical method for infrarenal or iliac infected aneurysms to avoid graft placement in contaminated areas. However, recently, extra-anatomic reconstruction has been indicated in cases with severe infections with pus or suppurative tissue around infrarenal or iliac aneurysms, while in-situ reconstruction is indicated in cases with low-grade infections and indications such as pseudoaneurysm or absence of pus [20]. In addition, in-situ reconstruction is indicated in cases of suprarenal or thoracoabdominal infected aneurysms, because of the anatomical difficulty of revascularization [20]. In our case, ampicillin administration based on the rapid diagnostic testing contributed to the early clearance of the microorganism from the blood. Therefore, we could perform in-situ reconstruction with revascularization and omental implantation based on the negative blood culture results, which suggested that there was probably no severe infection within the aneurysmal field.

In conclusion, there is no universally accepted strategy for diagnosing and treating *L. monocytogenes*-infected aneurysms; therefore, the therapeutic approach needs to be tailored individually. Rapid diagnostic testing can be a guide for devising therapeutic strategies for emergent infections, such as *L. monocytogenes*-infected aneurysms. We believe that rapid diagnostic testing should be performed in cases where Gram-positive rods are observed in blood cultures, especially from immunocompromised patients with infected aneurysms. *L. monocytogenes* is associated with prosthetic graft infections; therefore, extra-anatomic reconstruction is indicated in most cases of *L. monocytogenes*-infected iliac aneurysms. However, in-situ reconstruction can also be proposed in cases where appropriate intensive antibiotic therapy is administered, as in our case. We believe that appropriate and timely microbiological examinations, in addition to radiographic examinations, aid in deciding the optimal surgical procedures for individual patients, which may ensure preferable outcomes.

## Funding

This research did not receive any specific grant from funding agencies in the public, commercial, or not-for-profit sectors.

## Ethical approval

This study has been approved by the institutional review board and ethics committee of Japanese Red Cross Ise Hospital (Approval number: ER2020-87).

## Consent statement

Written informed consent was obtained from the patient for publication of this case report and accompanying images. A copy of the written consent is available for review by the Editor-in-Chief of this journal on request.

## CRediT authorship contribution statement

**Hirokazu Toyoshima:** Conceptualization, Methodology, Data curation, Writing - original draft, Writing - review & editing, Visualization. **Koji Hirano:** Methodology, Data curation. **Motoaki Tanigawa:** Supervision. **Naoto Masuda:** Conceptualization, Methodology. **Chiaki Ishiguro:** Conceptualization, Methodology, Supervision. **Hiroyuki Tanaka:** Methodology. **Yuki Nakanishi:** Methodology. **Shigetoshi Sakabe:** Supervision.

## Declaration of Competing Interest

The authors report no declarations of interest.
